# Flowering Time Gene Variation in *Brassica* Species Shows Evolutionary Principles

**DOI:** 10.3389/fpls.2017.01742

**Published:** 2017-10-17

**Authors:** Sarah V. Schiessl, Bruno Huettel, Diana Kuehn, Richard Reinhardt, Rod J. Snowdon

**Affiliations:** ^1^Department of Plant Breeding, IFZ Research Centre for Biosystems, Land Use and Nutrition, Justus Liebig University, Giessen, Germany; ^2^Max Planck Institute for Breeding Research, Cologne, Germany

**Keywords:** sequence capture, natural variation, polyploidy, speciation, copy number variation

## Abstract

Flowering time genes have a strong influence on successful reproduction and life cycle adaptation. However, their regulation is highly complex and only well understood in diploid model systems. For crops with a polyploid background from the genus *Brassica*, data on flowering time gene variation are scarce, although indispensable for modern breeding techniques like marker-assisted breeding. We have deep-sequenced all paralogs of 35 *Arabidopsis thaliana* flowering regulators using Sequence Capture followed by Illumina sequencing in two selected accessions of the vegetable species *Brassica rapa* and *Brassica oleracea*, respectively. Using these data, we were able to call SNPs, InDels and copy number variations (CNVs) for genes from the total flowering time network including central flowering regulators, but also genes from the vernalisation pathway, the photoperiod pathway, temperature regulation, the circadian clock and the downstream effectors. Comparing the results to a complementary data set from the allotetraploid species *Brassica napus*, we detected rearrangements in *B. napus* which probably occurred early after the allopolyploidisation event. Those data are both a valuable resource for flowering time research in those vegetable species, as well as a contribution to speciation genetics.

## Introduction

The genus *Brassica* is highly diverse. It contains many phenotypically extremely different vegetable, turnip and oil crops, among them garden turnip, Chinese cabbage and Pak Choi (*Brassica rapa*) and cabbage, broccoli, cauliflower, Brussels sprouts, kale, kohlrabi and savoy (*Brassica oleracea*) (Paterson et al., 2001). Both species are also diploid progenitors of *B. napus*, which comprises rapeseed/ canola and rutabagas (Chalhoub et al., 2014). Adequate regulation of flowering and flowering time is crucial for crop production especially for leafy vegetable crops as in *B. rapa* and *B. oleracea*. Early bolting limits vegetable growth and can therefore severely decrease yield. On the other hand, complete inhibition of flowering interferes with seed production. Knowledge about the impact of flowering time gene variation is therefore crucial for successful vegetable breeding. In the model plant *Arabidopsis thaliana*, flowering time is set by expression of the gene *FLOWERING LOCUS T (FT)* (Srikanth and Schmid, 2011; Blümel et al., 2015). The expression of *FT* is negatively regulated by the transcriptional repressor *FLOWERING LOCUS C (FLC)* in interaction with other genetic factors from the vernalisation pathway, and positively regulated via the transcriptional activator *CONSTANS (CO)* in interaction with genetic factors from the photoperiod pathway and the circadian clock (Srikanth and Schmid, 2011; Blümel et al., 2015). Other pathways like the ambient temperature pathway, the age pathway, the sugar signaling pathway and the stress pathway are able to modulate the flowering response (Srikanth and Schmid, 2011; Blümel et al., 2015). All the same, vernalisation and day length have major effects on flowering time. Although *B. rapa* and *B. oleracea* are closely related to *A. thaliana*, their reaction to vernalisation is different. Whereas *A. thaliana* and *B. rapa* respond to seed vernalisation, *B. oleracea* requires plant vernalisation (Lin, 2005; Zhang et al., 2015) and is not responsive at early seedling stages. As in *A. thaliana*, annual (vernalisation-independent) and biennual (vernalisation-dependent) forms exist within both *Brassica* species (Camargo and Osborn, 1996). Foregoing studies have identified different orthologous copies of *FLC* and *FT* as strong candidates for flowering time regulation in *B. rapa* and *B. oleracea*, both in the absence and in the presence of vernalisation (Pires et al., 2004; Lin, 2005; Razi et al., 2008; Zhao et al., 2010; Li et al., 2013; Zhang et al., 2015). *Brassica rapa* has been found to carry 2 copies of *Bra.FT* (Zhang et al., 2015) (A02 and A07), 4 copies of *Bra.FLC* (Schranz et al., 2002) (A02, two copies on A03, A10) and 3 copies of *Bra.CO* (A01, A03, A10). In contrast, *B. oleracea* seems to carry 4 copies of *Bol.FT* (two copies on C02, C04, and C06), 5 copies of *Bol.FLC* (Razi et al., 2008)(one copy on C02, two copies on C03, two copies on C09) and 3 copies of *Bol.CO* (C01, C03, and C09). *Bra.FT.A07*, often referred to as *BrFT2*, was found to underlie a strong QTL for flowering time, possibly due to a transposon insertion in the mapping parent R-o-18 (Zhang et al., 2015). *Bra.FLC.A02*, also referred to as *BrFLC2*, was found to underlie QTLs for flowering time in different studies (Zhao et al., 2010; Xiao et al., 2013; Zhang et al., 2015), possibly due to a 57 bp InDel in the fourth exon and the forth intron of the gene, leading to a non-functional allele (Wu et al., 2012). Another *FLC* copy, *Bra.FLC.A10*, also referred to as BrFLC1, was associated to flowering time due to alternative splicing via variation in intron 6 (Yuan et al., 2009; Wu et al., 2012). A CO-like copy on A02 co-localized with a flowering QTL in a DH population derived from a Chinese cabbage and a rapid cycling line (Li et al., 2013). Different patterns of functional polymorphisms, including premature stop codons, non-synonymous SNPs and differential promotor structure have been found for *Bol.FLC* copies (Okazaki et al., 2007; Razi et al., 2008; Irwin et al., 2016). Both copies on C03 (formerly referred to as BoFLC3 and BoFLC5) as well as one copy on C09 (referred to as BoFLC1) were found to co-localize with flowering time QTL (Razi et al., 2008). A further copy, referred to as BoFLC2 or BoFLC4, was assumed to be a pseudogene located on C02 (Razi et al., 2008), but was found to underlie a QTL in a different study due to a 1 bp deletion (Okazaki et al., 2007) Copies of *Bol.CO* were also suggested as candidate genes for QTL in *B. oleracea*, for example, *Bol.CO.C09* (Okazaki et al., 2007). Most previous research has therefore focused on the central flowering regulators *FLC, FT*, and *CO*, whereas other genes which might modulate the flowering response have been largely ignored. In order to provide a more complete description of genetic variation in central flowering time genes, we deep-sequenced representatives of two *B. rapa* subspecies (L58, ssp. *parachinensis*, R-o-18, ssp. *tricolaris*) along with two different genotypes of *B. oleracea* ssp. *capitata* (BRA1398, Kashirka) for a set of flowering time genes, using a sequence capture approach followed by Illumina sequencing. The data allowed estimation of copy number and sequence variation including SNPs and InDels. All those sequence variants are potentially influential on the phenotype and therefore an interesting resource to vegetable breeders. Comparison to previous data from the same genes in the allopolyploid hybrid species *B. napus* (Schiessl et al., 2014, 2017a,b) provide new insight into the genetic history of *B. napus* and are discussed along with the sequence data from its diploid progenitors.

## Material and methods

### Plant material and DNA extraction

Two inbred *B. rapa* lines and two *B. oleracea* genotypes were used for the present study. The two *B. rapa* lines, both annuals, were L58, a caixin line (ssp. *parachinensis*) and R-o-18, a yellow sarson line (ssp. *tricolaris*). Both had been used as parents for DH populations before (Bagheri et al., 2012; Zhang et al., 2015). The two *B. oleracea* genotypes were the annual BRA1398 (*ssp. capitata convar. botrytis var. botrytis L*.) and the biennial Kashirka (ssp. *capitata*), a late flowering Siberian kale.

Leaf material from 4 week old plants grown in pots in the greenhouse was collected and immediately shock-frozen in liquid nitrogen. DNA was then extracted from grinded leaf material using a common CTAB protocol modified from Doyle (1990) as described before (Schiessl et al., 2014). DNA concentration was measured using a Qubit fluorometer (Qubit dsDNA BR assay kit, Life Technologies, Darmstadt, Germany) according to the manufacturer's protocol. DNA quantity and purity was further checked on 0.5% agarose gel (3 V/cm, 0.5xTBE, 120 min) stained with ethidium bromide.

### Target genes

The four samples were re-sequenced using targeted deep sequencing along with 280 *B. napus* genotypes as described elsewhere (Schiessl et al., 2017a,b). In brief, flowering time genes involving the most important flowering regulation pathways as known from *Arabidopsis thaliana* were checked for *Brassica* orthologs. Those included genes from the circadian clock *(CYCLING DOF FACTOR 1 (CDF1), EARLY FLOWERING 3 (ELF3), GIGANTEA (GI)*, and *ZEITLUPE (ZTL)*), the vernalisation pathway (*EARLY FLOWERING 7 (ELF7), EARLY FLOWERING IN SHORT DAYS (EFS), FLOWERING LOCUS C (FLC), FRIGIDA (FRI), SHORT VEGTATIVE PHASE (SVP), SUPPRESSOR OF FRIGIDA 4 (SUF4), TERMINAL FLOWER 2 (TFL2), VERNALISATION 2 (VRN2), VERNALISATION INSENSITIVE 3 (VIN3)*), the photoperiod pathway (*CONSTANS (CO), CRYPTO-CHROME 2 (CRY2), PHYTOCHROME A (PHYA), PHYTOCHROME B (PHYB)*) and gibberellin signaling (*GIBBERELLIN-3-OXIDASE 1 (GA3ox1)*), along with downstream signal transducers (*AGAMOUS-LIKE 24 (AGL24), APETALA 1 (AP1), CAULIFLOWER (CAL), FLOWERING LOCUS D (FD), FLOWERING LOCUS T (FT), FRUITFUL (FUL), LEAFY (LFY), SQUAMOSA PROMOTOR PROTEIN LIKE 3 (SPL3), SUPPRESSOR OF CONSTANS 1 (SOC1), TEMPRANILLO 1 (TEM1), TERMINAL FLOWER 1 (TFL1)*). On top, we also included 6 further genes: *CIRCADIAN CLOCK ASSISTED 1 (CCA1), FLAGELLIN-SENSITIVE 2 (FLS2), GLYCIN-RICH PROTEIN 7 (GRP7), GLYCIN-RICH PROTEIN 8 (GRP8), GORDITA (GORD)* and *SENSITIVITY TO RED LIGHT REDUCED 1 (SRR1)*, giving a total of 35 genes.

### Bait development

In order to enrich for the respective target regions, a bait pool was constructed based on selected sequences from *B. rapa, B. oleracea*, and *B. napus*. A detailed description of the bait pool development can be found in (Schiessl et al., 2017a). The bait pool consisted of 178 bait groups, 63 bait groups for *B. rapa* orthologs, 71 bait groups for *B. oleracea* orthologs and 24 bait groups for *B. napus* orthologs. As a short summary, baits were first developed in the program eArrayXD using sequences from *B. rapa* and *B. oleracea*. After a preliminary sequencing test with four diverse *B. napus* genotypes (Schiessl et al., 2014), the bait pool was refined and some sequences were replaced by *B. napus* sequences using the Agilent Genomic Workbench program SureDesign (Agilent Inc., Santa Clara, CA, USA). This improved the specificity of the bait pool (Schiessl et al., 2017a). Bait groups were created using the “Bait Tiling” tool. The parameters were set as follows: Sequencing Technology: “Illumina,” Sequencing Protocol: “Paired-End long Read (75 bp+),” “Use Optimized Parameters (Bait length 120, Tiling Frequency 1x),” Avoid Overlap: “20,” “User defined genome,” “Avoid Standard Repeat Masked Regions.”

### Library preparation and sequencing

Custom bait production was carried out by Agilent Technologies (Agilent Inc., Santa Clara, CA, USA) using the output oligonucleotide sequences from SureDesign. Sequence capture was performed at the GenomeCenter at the Max Planck Institute for Breeding Research (Cologne, Germany) using the SureSelectXT 1–499 kb Custom Kit (Agilent Inc., Santa Clara, CA, USA) according to the manufacturer's instructions. The resulting TruSeq DNA library (Illumina Inc., San Diego, CA, USA) was sequenced on an Illumina HiSeq 2500 sequencer at the Max Planck Institute for Breeding Research (Cologne, Germany) in 100 bp single read mode.

### Sequence analysis

#### Alignment

Quality control of the raw sequencing data was performed using FASTQC. Reads were mapped both onto version 4.1 of the *B. napus* “Darmor-Bzh” reference genome sequence assembly (Chalhoub et al., 2014) and either onto version 1.5 of the *B. rapa* “Chiifu-401-42” reference genome (Wang et al., 2011) for *B. rapa* reads or onto version 2.1 “TO1000” of the *B. oleracea* reference genome (Parkin et al., 2014) for *B. oleracea* reads. Mapping was performed using the SOAPaligner algorithm (Li R. et al., 2009), with default settings. Removal of duplicates, sorting and indexing was carried out with samtools version 0.1.19 (Li H. et al., 2009). Alignments were visualized using the IGV browser version 2.3.12 (Robinson et al., 2011). For InDel calling, a separate mapping using Bowtie2 (Langmead and Salzberg, 2012) was performed, as described in (Schmutzer et al., 2015), on the reference genome of *B. rapa* and *B. oleracea*. Removal of duplicates, sorting and indexing was again carried out with samtools version 0.1.19.

As it turned out that *Bol.FLC.C02* is likely to be misassembled in the reference genome, we cut out all *Bol.FLC* copies from the reference genome except *Bol.FLC.C02*, which we replaced by *Bol.FLC.C2.E9* (GenBank accession KU521323.1 Irwin et al., 2016). The resulting fasta file was used as artificial genome and the mapping was performed accordingly.

#### CNV calling

We first defined regions with sufficient coverage (normalized mean coverage at least 10) for *B. rapa* and *B. oleracea* mapped on their respective reference genomes. A region is defined as being covered by at least two overlapping reads. The coverage was calculated using the bedtools software with multiBamCov (Quinlan, 2014) and normalized to region length, genotype read number and genome size. Those regions were subjected to BLAST against the target regions found for *B. napus* in (Schiessl et al., 2017a) using a *e*-value cut-off of e^−50^ using the program BioEdit version 7.2.0. Moreover, a bed file with the positions of those regions was compared to the gene positions of the respective reference genomes. All regions which either overlapped with an annotated gene or alternatively had a BLAST hit to a *B. napus* target region were analyzed for CNVs. In order to have comparable coverages, we used the gene positions wherever possible, and calculated the normalized coverages on those positions.

In a second step, we compared the coverage ratio between both genotypes of a species. If one genotype had less than 50% coverage than the other, we assumed an unbalanced coverage ratio, indicating a CNV. In case that the coverage of one of the genotypes was less than 30% of the other, we assumed a deletion. For all other cases, we compared the coverage of this region to the respective coverage obtained for the orthologous region in a population of 280 *B. napus* genotypes (Schiessl et al., 2017a). If the coverage ratio was less than 30%, we assumed a deletion. In all other cases, we assumed a duplication for the other genotype.

#### SNP and InDel calling and annotation

Calling of single nucleotide polymorphisms (SNPs) was performed with the algorithm mpileup in the samtools toolkit (Li H. et al., 2009). Calling of InDels was performed based on a separate alignment using Bowtie2. An initial InDel calling was first performed using samtools mpileup, and realignment of reads around InDels was then performed using GATK RealignerTargetCreator, version 3.1.1 (McKenna et al., 2010). A final InDel calling was then performed as described above. SNPs were filtered for a minimum mapping quality of 50 and a read depth of at least 10, and InDels were filtered for a minimum mapping quality of 30 and a read depth of at least 10 using vcftools (Danecek et al., 2011). SNP and InDel annotation was performed using CooVar (Vergara et al., 2012).

As InDel calling with this read length and mapping parameters is limited to InDels of a length of 18 bp, we conducted another approach where we searched for regions of zero coverage in a 19 bp window which were strongly covered in the respective other genotype using the SOAP2 mapping, while having a low coverage using the realigned Bowtie2 mapping in the same genotype. This approach ensures for the detection of larger deletions, which are not due to reference mapping problems.

### Data and seed availability

The aligned bamfiles are available via NCBI SRA (https://www.ncbi.nlm.nih.gov/sra/), study accession SRP119226 (Flowering time genes in *B. rapa* and *B. oleracea*).

The two accessions of *B. oleracea* BRA1398 and Kashirka (BRA1506) are available via the Genbank at IPK Gatersleben. The two accessions of *B. rapa* L58 and R-o-18 are available from the authors upon request.

## Results

### Gene copies in *B. rapa, B. oleracea*, and *B. napus*

In the two *B. rapa* accessions we found 1405 regions with a mean normalized coverage of at least 10, among them 222 regions co-localizing with an annotated *B. rapa* gene. Of those, 105 regions had a BLAST hit to a target gene of *B. napus* as analyzed in (Schiessl et al., 2017b), while 95 regions of those with a BLAST hit co-localized with an annotated *B. rapa* gene. We therefore analyzed 228 regions for *B. rapa*, which either co-localized with a *B. rapa* gene or had a BLAST hit to a target *B. napus* gene or both, excluding non-genic regions from the analysis. For the two *B. oleracea* accessions we found 3010 regions with a normalized coverage of at least 10, with 365 regions co-localizing with an annotated *B. oleracea* gene and 111 regions showing a BLAST hit to a target *B. napus* region. In total, we analyzed 384 regions for *B. oleracea*.

**Figure 1:**
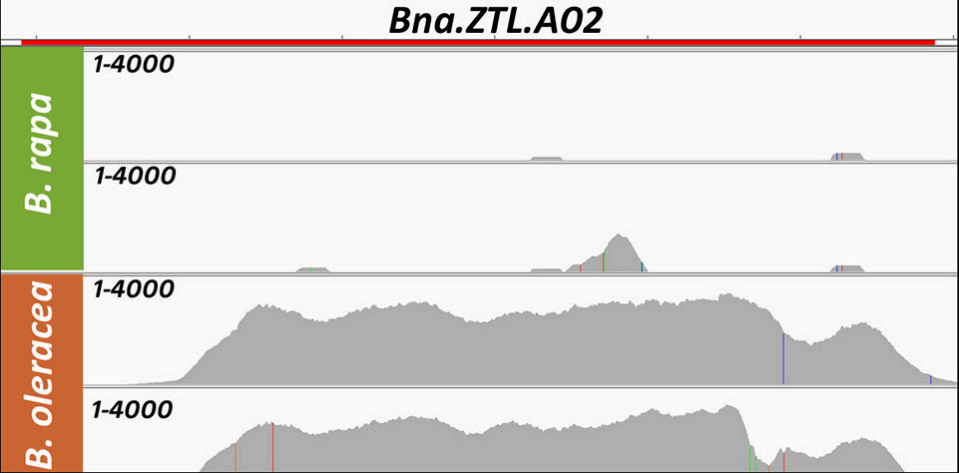
Coverage (Raw read depth) for both genotypes of *B. rapa* (green, A subgenome) showing low coverage and both genotypes of *B. oleracea* (orange, C subgenome) showing high coverage when mapped on the *B. napus* genome for a gene located on the A subgenome in *B. napus*: *Bna.ZTL.A02* (*BnaA02g16710D*) as an example for early rearrangements in the *B. napus* genome. The height of the gray area in each lane marks the raw read depth and is shown from 1 to 4000 as read count per base as non-dimensional number. The red bar on top marks the gene extension.

**Figure 2:**
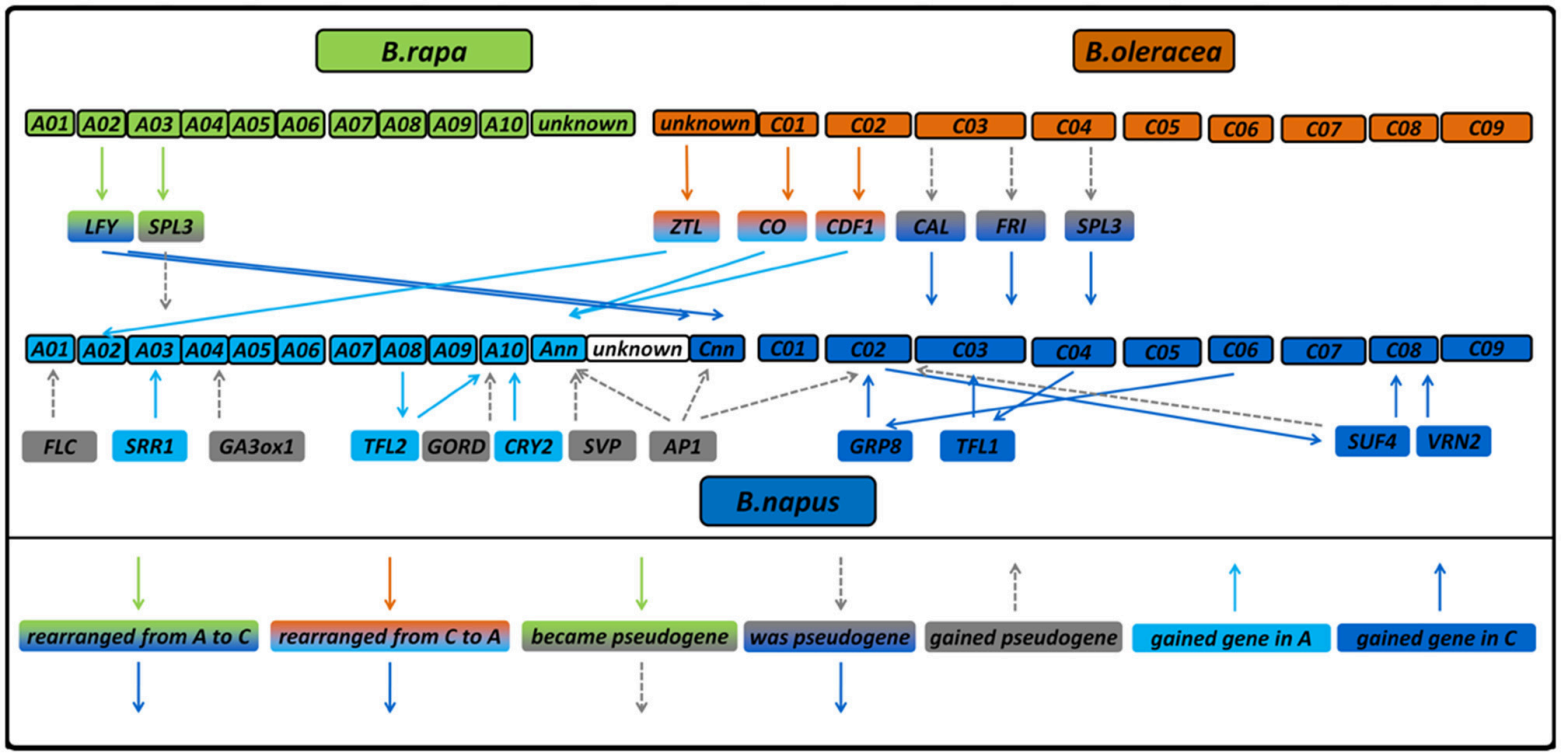
Diagram showing gene copies which were lost, gained or rearranged in *B. napus* compared to its diploid progenitors. The diagram also shows genetic regions which were considered to be pseudogenes in *B. napus* before (Schiessl et al., 2017a) without having a homologous region in the diploids, those which have a homologous region and those which were not annotated as a gene in the diploids. Gene copies without changes are not displayed.

When mapping the sequencing reads from *B. rapa* and *B. oleracea* onto the *B. napus* reference genome, we found that *BnaA02g16710D* (*Bna.ZTL.A02*) had a strongly reduced normalized coverage in both *B. rapa* lines (2 and 13% of the mean *B. napus* coverage), while both *B.oleracea* lines had significant coverage at this locus (169 and 159% of the mean *B. napus* coverage). The raw read depth landscape for this locus is shown in Figure 1. The *B. rapa* genome also did not carry a respectively annotated gene on A02, while the *B. oleracea* genome carries an additional copy on a non-localized scaffold (Figure 2).

In contrast, *BnaCnng78500D* (*Bna.LFY.Cnn*) showed a much higher coverage in both *B. rapa* lines (270 and 256% of the mean *B. napus* coverage) than in both *B. oleracea* (41 and 45% of the mean *B. napus* coverage). Because the total size of the sequenced gene space was about half the size of the allotetraploid while the read number was comparable, a normalized coverage increase of around 200% would be expected for the diploid species.

**Figure 3:**
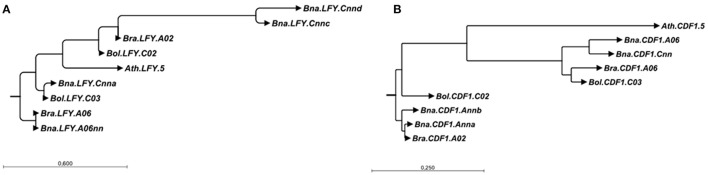
Neighbor-joining trees for all copies of *LFY* **(A)** and *CDF1* **(B)** in *B. rapa, B. oleracea* and *B. napus* using the *A. thaliana* gene as outgroup. The trees were constructed based on aligments made in CLC SequenceViewer version 7.8.1 with bootstrap analysis (100 replicates).

In contrast to *B. napus*, we found no orthologs to the four further gene copies *BnaA03g24400D* (*Bna.SRR1.A03*), *BnaA10g27730D* (*Bna.CRY2.A10b*), *BnaAnng24480D* (*Bna.CDF1.Ann*) and *BnaAnng38870D* (*Bna.CO.Annb*) in *B. rapa*. All the same, when mapping the *B. rapa* reads onto the *B. napus* genome, we found significant coverage on those loci (176, 123, 207, and 87% of the *B. napus* coverage, respectively). This might either indicate mismapping or missing information in the reference genome. Doing so for *B. oleracea*, we did not find orthologs to *BnaC08g10770D* (*Bna.VRN2.C08*) and *BnaCnng50250D* (*Bna.LFY.Cnna*). The last one could point to a stable exchange between the A and C subgenomes similar to *BnaCnng78500D* (*Bna.LFY.Cnnb*), because it had only 51% coverage with *B. oleracea* reads, while having 136% using *B. rapa* reads. This is further supported when looking at the neighbor-joining tree for LFY (Figure 3A). On the other hand, we also noticed that the *B. napus* sequence of *BnaCnng50250D (Bna.LFY.Cnna)* contained patches of NNN (unknown sequence), so is possibly an artifact of the *B. napus* reference genome and might also interfere with the mapping for *BnaCnng78500D* (*Bna.LFY.Cnnb*). *Bna.VRN2.C08* had 173% using *B. oleracea* reads. For *Bol.CDF1.C02*, we found a strongly covered region without any *B. napus* ortholog, which could indicate a rearrangement from *Bol.CDF1.C02* to *Bna.CDF1.Ann* (see also Figure 3B). An overview of all putative gains and losses can be found in Figure 2. *BnaC02g00490D* (*Bna.FLC.C02*), showing 192% coverage using *B. oleracea* reads was not found to be covered at the respective locus *Bo2g166560 (Bol.FLC.C02*) in *B. oleracea* itself in both lines. We assume that this is due to a misassembled copy in the reference genome in *B. oleracea*, due to phylogenetic analysis and comparisons to sequences for *Bol.FLC.C2* published by (Irwin et al., 2016). When we performed a separate alignment only to the *Bol.FLC* copies replacing *Bol.FLC.C02* by the published version in (Irwin et al., 2016), we got a coverage in the expected range on *Bol.FLC.C2.E9*. Our data suggest that there is a misassembly between *Bol.FLC.C02* and *Bol.FLC.C03a*.

### Copy number variation

For all analyzed regions which co-localized with a respectively annotated gene, we analyzed the coverage for the annotated gene positions. For those which only had a BLAST hit to a target *B. napus* gene, we analyzed the coverage for the total length of the covered region.

For *B. rapa*, we found 61 regions with an unbalanced coverage ratio, meaning that the coverage for one of the genotypes was at least 50% higher than for the other. Of those, 55 regions were genic in *B. rapa*. While 13 genic regions showed clear deletions (one genotype had less than 30% normalized coverage compared to the other), the coverage patterns of the other regions were less obvious. Therefore, in order to distinguish between a duplication in one genotype and a deletion in the other, we compared the normalized coverages of the *B. rapa* region to the respective normalized mean coverage of the corresponding region in a population of 280 genotypes of *B. napus* (Schiessl et al., 2017b). All regions with less than 30% coverage compared to *B. napus* were considered to be deleted. All other unbalanced coverage ratios were assigned as duplication to the respective genotype. According to this definition, we found 15 genic deletions and 25 genic duplications in the genotype L58, and 9 genic deletions and 10 duplications in the genotype R-o-18. The CNVs concerning target genes are summarized in Table 1.

**Table 1:** Gene names, Gene IDs, chromosomal location and normalized coverages for target flowering genes for the two sequenced accessions of *B. rapa*.

**Target gene**	**Gene ID *B. napus***	**Gene ID *B. rapa***	**Chromosome *B. rapa***	**Mean normalized coverage *B. napus* population**	**Mean normalized coverage *B. rapa* on *B. napus***	**Mean normalized coverage *B. rapa* on *B. rapa***	**Normalized coverage genotype L58 on *B. napus***	**Normalized coverage genotype L58 on *B. rapa***	**Assumed nature of the CNV**
*Bna.VIN3.A02*	*BnaA02g08140D*	*Bra020445*	A02	1,561.9	4,982.2	1,441.3	6,330.0	1,833.2	Duplication
*Bna.TEM1.A02*	*BnaA02g14040D*	*Bra038346*	A02	1,281.5	2,111.5	702.6	2,637.1	884.7	Duplication
*Bna.PHYB.A03*	*BnaA03g34390D*	*Bra001650*	A03	1,637.4	3,159.4	1,076.7	3,986.9	659.3	Deletion
*Bna.SVP.A04*	*BnaA04g12990D*	*Bra030228*	A04	1,196.7	2,485.7	960.0	3,246.9	1,238.4	Duplication
*Bna.CRY2.A08*	*BnaA08g27870D*	*Bra030568*	A08	1,195.9	1,769.3	950.9	1,788.6	712.5	Deletion
*Bna.SUF4.A09*	*BnaA09g25530D*	*Bra023153*	A09	1,467.6	2,513.5	1,249.9	2,506.9	1,512.6	Duplication
*Bna.GA3ox.A09*	*BnaA09g57140D*	*Bra026757*	A09	1,675.6	1,114.2	594.5	720.1	425.4	Deletion
*Bna.CO-li.A10*	*BnaA10g18420D*	*Bra008668*	A10	2,248.3	3,936.3	1,398.6	4,418.0	994.0	Deletion
*Bna.SUF4.Ann*	*BnaAnng11220D*	*Bra039880*	A08	1,050.3	2,338.2	1,042.0	1,600.3	1,261.8	Duplication
*Bna.CO.Ann*	*BnaAnng39160D*	*Bra021464*	A01	795.4	2,212.1	1,009.1	3201.7	1,304.3	Duplication
**Target gene**	**Gene ID** ***B. napus***	**Gene ID** ***B. rapa***	**Chromosome** ***B. rapa***	**Mean normalized coverage** ***B. napus*** **population**	**Mean normalized coverage** ***B. rapa*** **on** ***B. napus***	**Mean normalized coverage** ***B. rapa*** **on** ***B. rapa***	**Normalized coverage genotype R-0-18 on** ***B. napus***	**Normalized coverage genotype R-0-18 on** ***B. rapa***	**Assumed nature of the CNV**
*Bna.PHYA.A06*	*BnaA06g05470D*	*Bra020013*	A06	1,104.8	1,423.0	826.4	1,521.4	1,077.1	Duplication
*Bna.ZTL.A07*	*BnaA07g01230D*	*Bra038830*	A07	1,265.0	3,121.8	1,343.2	2,839.7	1,627.4	Duplication
*Bna.EFS.A07*	*BnaA07g33460D*	*Bra015678*	A07	1,171.4	2,417.1	945.9	3,089.6	1,179.9	Duplication
*Bna.EFS.A07*	*BnaA07g33460D*	*Bra015678*	A07	1,171.4	2,417.1	945.9	3,089.6	1,179.9	Duplication
*Bna.ELF7.A10*	*BnaA10g27050D*	*Bra009582*	A10	1,094.2	2,348.5	1,120.3	2,609.5	1,386.0	Duplication
*Bna.TFL1.Ann*	*BnaAnng00810D*	*Bra028815*	A02	1,576.4	2,931.8	1,266.9	3,435.0	1,542.4	Duplication
*Bna.GRP7.Ann*		*Bra031210*	A09	322.3	670.1	510.3	594.5	700.5	Duplication

For *B. oleracea*, we found 118 regions with an unbalanced coverage ratio, with 110 regions being genic in *B. oleracea*. Applying the same thresholds as for *B. rapa*, we found 8 genic deletions and 38 genic duplications for genotype BRA1398, as well as 11 genic deletions and 46 duplications for genotype Kashirka. The respectively concerned target genes are summarized in Table 2.

**Table 2:** Gene names, Gene IDs, chromosomal location and normalized coverages for target flowering genes for the two sequenced lines of *B. oleracea*.

**Target gene**	**Gene ID *B. napus***	**Gene ID *B.oleracea***	**Chromosome *B.oleracea***	**Mean normalized coverage *B. napus* population**	**Mean normalized coverage *B. oleracea* on *B. napus***	**Mean normalized coverage *B. oleracea* on *B. oleracea***	**Normalized coverage genotype BRA1398 on *B. napus***	**Normalized coverage genotype BRA1398 on *B.oleracea***	**Assumed nature of the CNV**
*Bna.FT.C02*	*BnaC02g23820D*	*Bo01129s030*	Scaffold01129	95.2	69.3	51.4	0.5	0.6	Deletion
*Bna.FRI.C03*	*BnaC03g16130D*	*NA*	C3	1,092.3	1,965.8	NA	1,721.2	NA	Duplication
*Bna.FT.C06*	*BnaC06g27090D*	*Bo6g099320*	C6	490.3	888.6	1209.0	627.5	1,514.5	Duplication
*Bna.EFS.C06*	*BnaC06g38010D*	*Bo6g121240*	C6	1,379.9	2,242.1	1,481.9	3,276.3	2,014.1	Duplication
*Bna.SUF4.C08*	*BnaC08g09340D*	*Bo2g121020*	C2	1,321.7	4,003.0	2,995.4	5,437.6	4,023.6	Duplication
*Bna.PHYB.C08*	*BnaC08g10540D*	*Bo8g043460*	C8	76.8	45.9	27.6	0.3	0.2	Deletion
**Target gene**	**Gene ID** ***B. napus***	**Gene ID** ***B.oleracea***	**Chromosome** ***B.oleracea***	**Mean normalized coverage** ***B. napus*** **population**	**Mean normalized coverage** ***B. oleracea*** **on** ***B. napus***	**Mean normalized coverage** ***B. oleracea*** **on** ***B. oleracea***	**Normalized coverage genotype Kashirka on** ***B. napus***	**Normalized coverage genotype Kashirka on** ***B. oleracea***	**Assumed nature of the CNV**
*Bna.AP1.C02*	*BnaC02g44500D*	*Bo2g062650*	C2	1,635.1	1,891.5	2,082.4	3,040.8	3,126.3	Duplication
*Bna.TFL1.C03*	*BnaC03g47080D*	*Bo3g040460*	C3	904.5	1,028.9	752.8	1,522.7	1,055.7	Duplication
*Bna.CAL.C03*	*BnaC03g56640D*	*Bo00825s090*	Scaffold00825	822.7	842.2	1,237.0	1,206.9	1,718.7	Duplication
*Bna.TFL1.C04*	*BnaC04g16750D*	*Bo4g074330*	C4	1,290.8	2,202.5	1,403.5	1,850.2	1,742.2	Duplication
*Bna.SRR1.C09*	*BnaC09g34850D*	*Bo9g139490*	C9	1,087.5	1,649.7	1,324.3	1,243.6	1,669.0	Duplication
*Bna.CO.C09*	*BnaC09g41990D*	*Bo9g163730*	C9	1,466.0	1,103.9	1,004.9	3.2	2.7	Deletion
*Bna.FLC.C09*	*BnaC09g46540D*	*Bo9g173400*	C9	1,031.5	4,037.8	1,523.2	4,302.0	1,885.6	Duplication
*Bna.VRN2.Cnn*	*BnaCnng45490D*	*Bo5g078770*	C5	207.0	253.1	155.1	337.3	227.1	Duplication

### SNPs and indels

**Figure 4:**
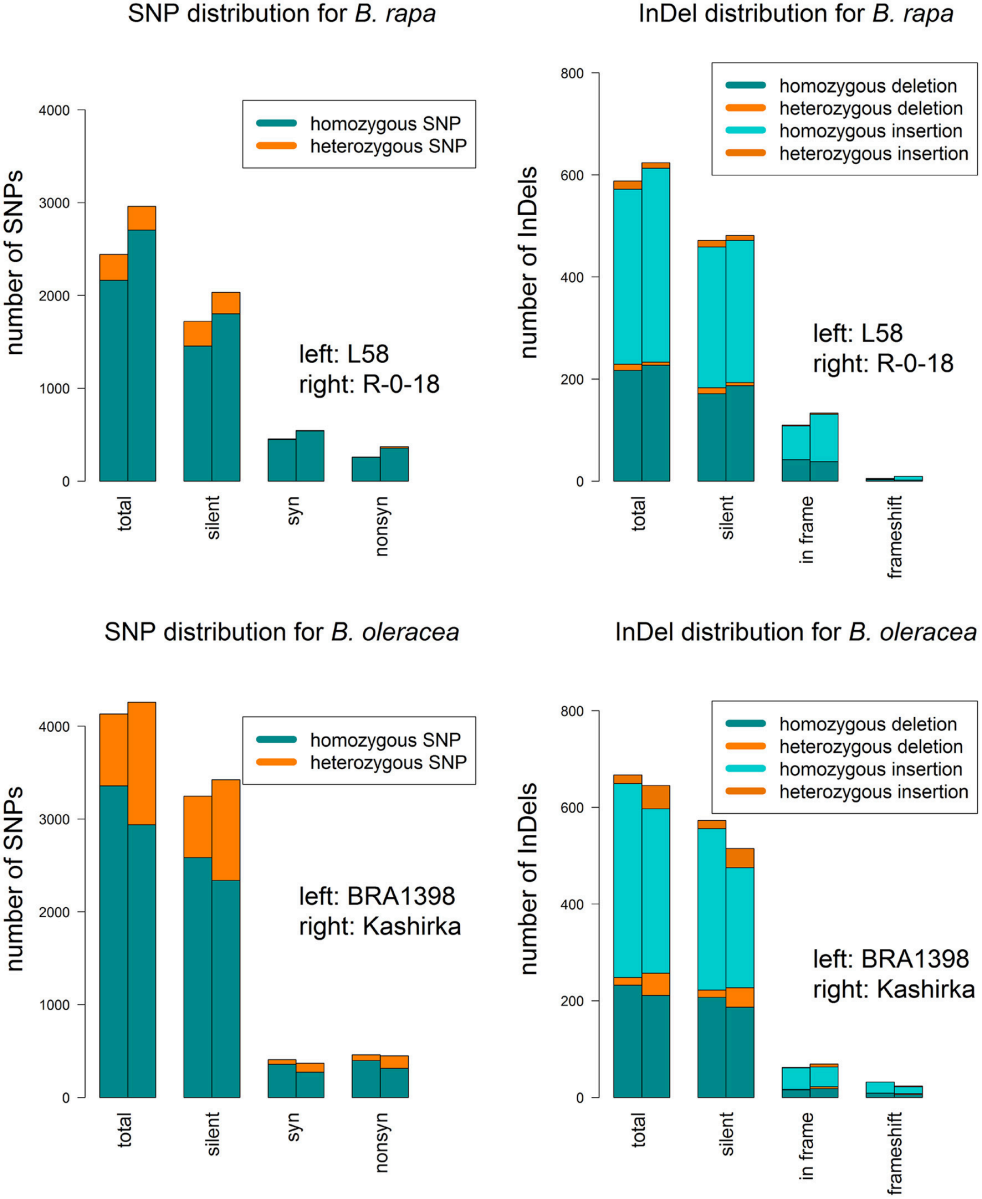
SNP and InDel distribution for both sequenced genotypes for each *B. rapa* and *B.oleracea*. For SNPs, the barplots show the total number of SNPs (total) and their distribution on silent SNPs (silent), synonymous SNPs (syn) and non-synonymous SNPs (non-syn). For InDels, they show the total number of InDels (total) and their distribution on silent InDels (silent), in-frame variants (in frame) and frameshift InDels (frameshift). Splice variants were not displayed, as they were rare. Homozygous and heterozygous SNPs are color-coded.

**Figure 5:**
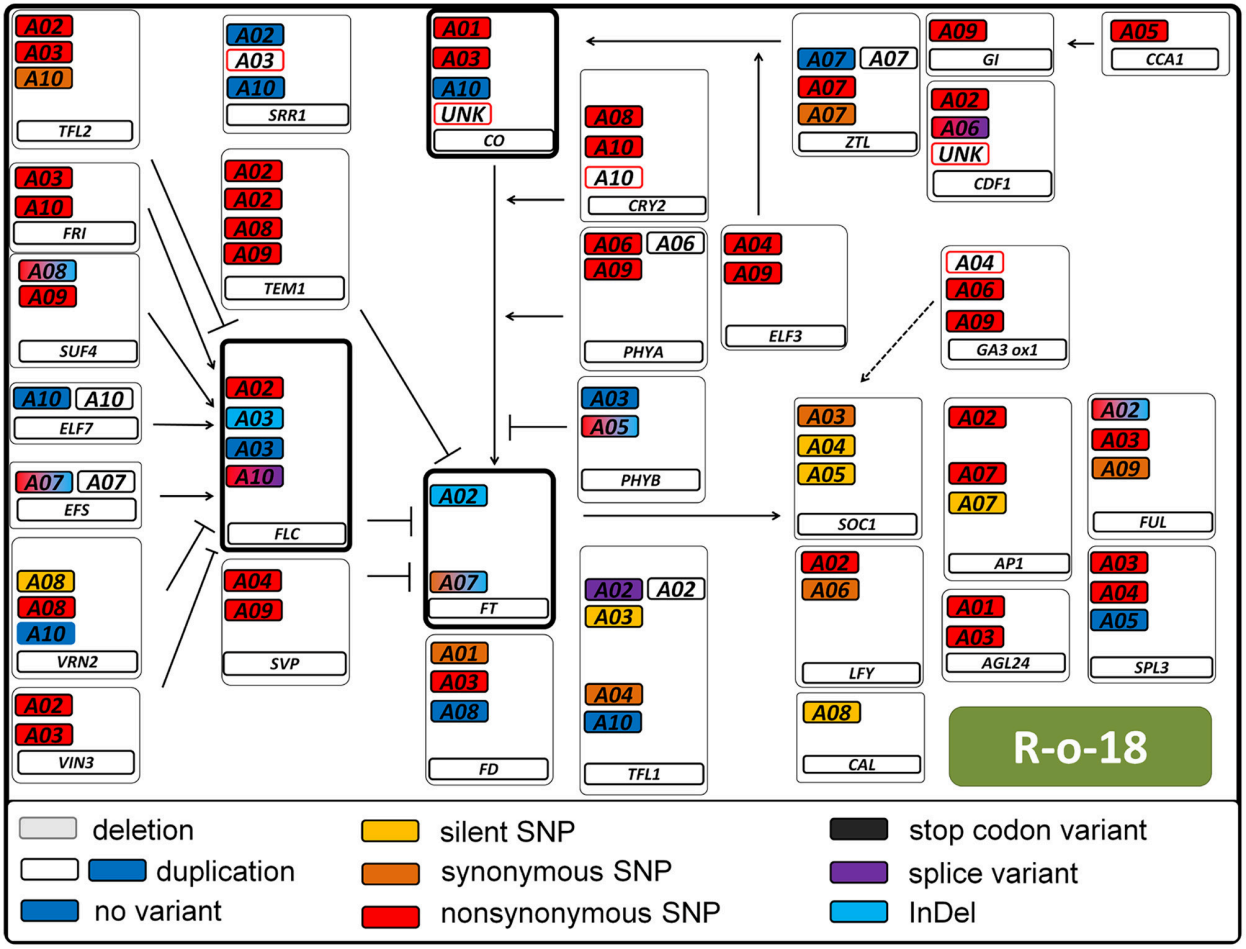
Pathway variation for flowering time genes in the *B. rapa* genotype R-o-18. Interactions are displayed as known from *Arabidopsis thaliana*. Arrows indicate positive regulation, blunt ends indicate negative regulation. All copies of a gene are displayed in a box. The main flowering regulators *FT, FLC*, and *CO* are indicated in bold boxes. The type of variant is color coded (see legend). Duplications are indicated by a second colorless copy box. SNP colors are hierarchic, meaning for example that synonymous SNPs are not displayed if non-synonymous SNPs are present. Boxes framed in red indicate known copies from *B. napus* which were not found in *B. rapa*. Boxes framed in blue were found, but not annotated as gene.

**Figure 6:**
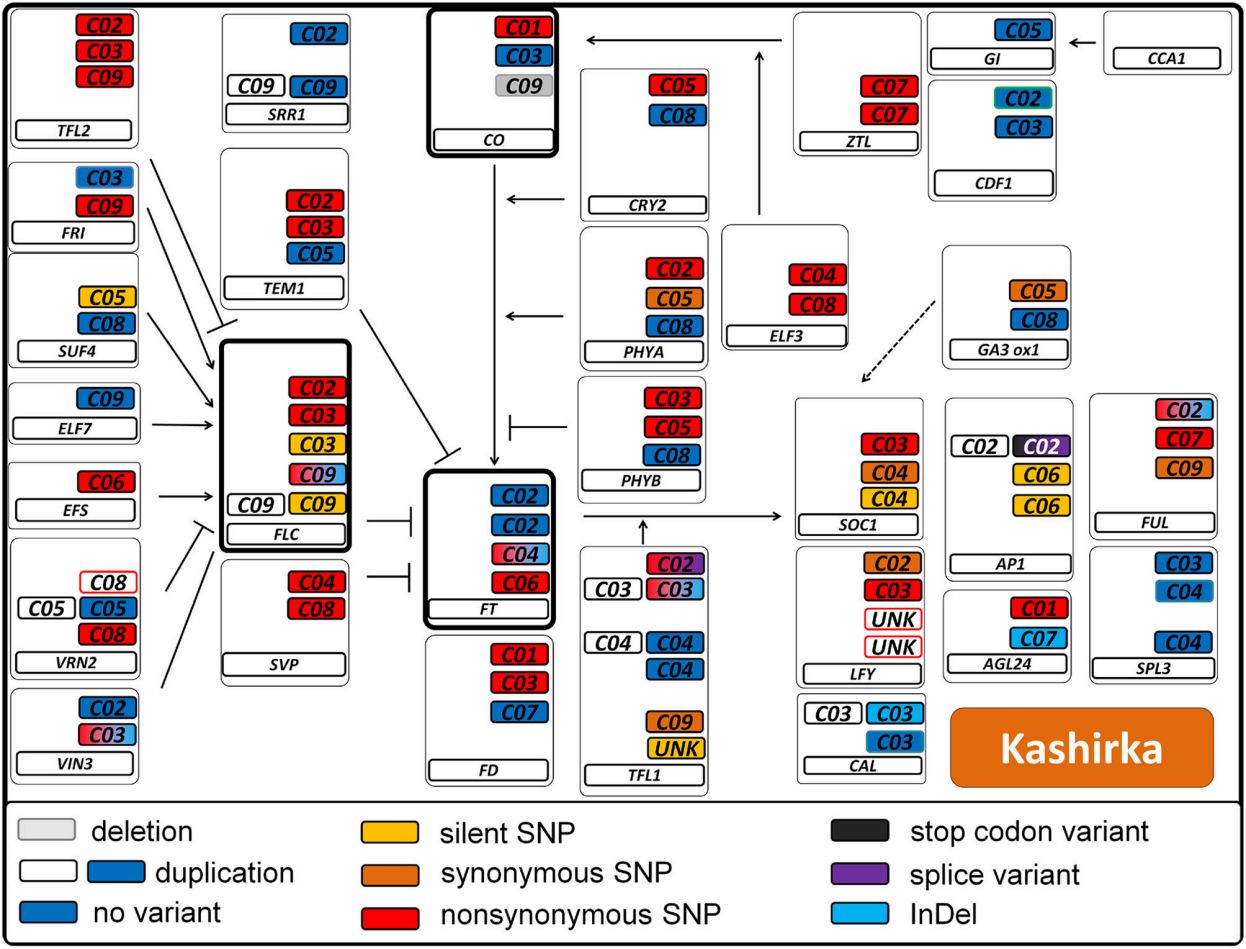
Pathway variation for flowering time genes in the *B. oleracea* genotype Kashirka. Interactions are displayed as known from *Arabidopsis thaliana*. Arrows indicate positive regulation, blunt ends indicate negative regulation. All copies of a gene are displayed in a box. The main flowering regulators *FT, FLC*, and *CO* are indicated in bold boxes. The type of variant is color coded (see legend). Duplications are indicated by a second colorless copy box. SNP colors are hierarchic, meaning for example that synonymous SNPs are not displayed if non-synonymous SNPs are present. Boxes framed in red indicate known copies from *B. napus* which were not found in *B. oleracea*. Boxes framed in green indicate that this copy was not known from *B. napus*. Boxes framed in blue were found, but not annotated as gene.

We called a total of 4409 SNPs and 1048 short InDels for *B. rapa* and 6743 SNPs and 1092 short InDels for *B. oleracea*. For *B. rapa*, 11.1% of the total SNPs and 4.3% of the total InDels were heterozygous, while 27.6% of the total SNPs and 11.4% of the total InDels were heterozygous for *B. oleracea*. As shown in Tables S1, S2, 1436 SNPs and 22 InDels were target variants for *B. rapa*, while 1179 SNPs and 22 InDels were target variants for *B. oleracea*. Taking SNPs and InDels together, the heterozygosity in *B. rapa* was 0.8% in the target regions (1.0% for L58 and 0.6% for R-o-18) and 15.7% for *B. oleracea* (5.2% for BRA1398 and 20.2% for Kashirka). The higher level of heterozygosity in *B. oleracea* was expected, as the species has a high level of self-incompatibility. The heterozygosity between both lines also varied more for *B. oleracea* than for *B. rapa* (Figure 4). Only homozygous variants were considered as true variants and used for further analysis. The variant distribution is shown in Figure 5 (R-o-18) and in Figure 6 (Kashirka) (see Figures S1, S2 for L58 and BRA1398). Almost all gene copy groups showed considerable variation in all genotypes. For L58, gene copy groups without putative functional variation were *Bra.SUF4, Bra.ELF7*, and *Bra.SVP* from the vernalisation pathway and *Bra.AP1, Bra.CAL, Bra.LFY*, and *Bra.SOC1* from the effector network. For R-o-18, gene copy groups without putative functional variation were *Bra.ELF7* from the vernalisation pathway, and *Bra.CAL* and *Bra.SOC1* from the effector network. Concerning *B. oleracea*, genotype BRA1398, only *Bol.AP1* and *Bol.SPL3* from the effector network remained without putative functional variation, while Kashirka did not show putative functional variation for *Bol.SUF4* and *Bol.ELF7* from the vernalisation pathway, for *Bol.GI* and *Bol.CDF1* from the photoperiodic pathway, for *Bol.GA3ox1* from GA signaling and for *Bol.SPL3* from the effector network.

As InDels larger than 18 bp were not detected with our read length and mapping parameters, we used an additional approach to detect larger deletions (see section Materials and Methods). For *B. rapa*, we found larger deletions for one gene copy in L58 (*Bra.PHYA.A06*) and for 5 copies in R-o-18 (among them *Bra.FLC.A02, Bra.TEM1.A02, Bra.SUF4.A08, Bra.CRY2.A10*). For *B. oleracea*, we did not find larger deletions in the annotated target genes.

### Variation in central flowering regulators

In *B. rapa*, the two copies of the central flowering regulator *FT* showed a low SNP variation. There was only one conservative L49I mutation in *Bra.FT.A02* in L58. However, there were 6 InDels in *Bra.FT.A02* (2 deletions, 4 insertions) for L58 and 2 deletions in the same copy in R-o-18 and an additional insertion into *Bra.FT.A07*. In contrast, there were 4 non-synonymous SNPs for *B. oleracea FT* copies. One of them was a radical W170C mutation in *Bol.FT.C04* in BRA1398, another a moderately radical H81Y mutation in the same copy in both genotypes. This copy also carried an insertion in both genotypes. The other two mutations, both found for *Bol.FT.C06* in Kashirka, were conservative (R21Q and E59D).

For *FLC* orthologs, there was more variation in *B. oleracea* than in *B. rapa*. There was a moderately radical R193P mutation and a deletion in *Bra.FLC.A02* in R-o-18 and a conservative T20P mutation along with a splice donor variant in *Bra.FLC.A10* for both genotypes, while the two copies on A03 remain almost conserved, with one insertion into *Bra.FLC.A03 (Bra022771*) shared by both genotypes. In contrast, the *B. oleracea* genotype BRA1398 shows variation in all *FLC* orthologs. Those were a moderately radical G110V mutation and a moderately conservative K79N mutation in *Bol.FLC.C03 (Bo3g005470*), a conservative I173V mutation shared with Kashirka in *Bol.FLC.C03 (Bo3g024250)*, a moderately radical T176N mutation also shared with Kashirka in *Bol.FLC.C09 (Bo9g173370*) and a conservative S168N mutation in *Bol.FLC.C09 (Bo9g173400)*. Both genotypes share an insertion in *Bol.FLC.C09 (Bo9g173370*). Kashirka also carries a conservative R24Q mutation in *Bol.FLC.C03 (Bo3g024250*) and putatively has *Bol.FLC.C09 (Bo9g173400*) duplicated. As *Bol.FLC.C09a* (*Bo9g173370*) seems to have an improper stop codon, producing a distinctly longer peptide, we assume that this copy is non-functional. As we found that *Bol.FLC.C02* appears to be misassembled in the *B. oleracea* genome, we called SNPs separately compared to *Bol.FLC.C2.E9*, a sequenced copy from genotype E9 published in (Irwin et al., 2016). We called one moderately radical A75D mutation in Kashirka compared to E9.

Orthologs of the key photoperiodic transcription factor *CO* show more variation in *B. rapa* than in *B. oleracea*. *Bra.CO.A01* shows two moderately radical (A60E, C237S), two moderately conservative (D16G, P130Q) and two conservative mutations (H167Q, Q181E) in R-o-18. In L58, this copy does not carry non-synonymous SNPs, but appears to be duplicated. *Bra.CO.A03* carries a radical F146S mutation, two moderately radical (A20D, Q92L) mutations, one moderately conservative E33G mutation and one conservative I192V mutation in both genotypes. Both *B. oleracea* genotypes carry a moderately radical Y100S mutation in *Bol.CO.C01*, while BRA1398 also carries two moderately radical mutations (K145I, G223R) and one conservative E71Q mutation on *Bol.CO.C03*. The copy *Bol.CO.C09* appears to be deleted in Kashirka.

## Discussion

### Network variation in vegetable species

Our sequencing data provide considerable novel data on variation among numerous flowering time regulatory genes in *B. rapa* and *B. oleracea*. We confirmed the functional conservation of *BrFLC2 (Bra.FLC.A02)* in the *B. rapa* genotype L58 (Wu et al., 2012) and a previously identified SNP resulting in a splice variant in *BrFLC1 (Bra.FLC.A10)* (Yuan et al., 2009; Wu et al., 2012). A larger deletion in *Bra.FLC.A02* in genotype R-o-18 colocalizes with a 57 bp deletion at the same position in intron 4 and exon 4. This deletion was previously found to underlie a flowering time QTL in a DH population deriving from L58 and R-o-18 (Zhang et al., 2015). We moreover observed an InDel in *BrFT2 (Bra.FT.A07)* in R-o-18, which we assume is caused by a larger structural variant underlying another flowering time QTL in the same population (Zhang et al., 2015). For *B. oleracea*, the variation detected in the central flowering time regulators is expected to significantly influence flowering time and related processes, considering the large genetic variation including radical SNP mutations, InDels and CNVs. QTL studies for flowering time in *B. oleracea* found central regulator copies in different populations (Okazaki et al., 2007; Razi et al., 2008; Irwin et al., 2016). Both *Bol.FLC.C03* and one *Bol.FLC.C09* copy were found to underlie flowering time variation, and *Bol.FLC.C02* variation has been found to have a large influence on heading date in purple sprouting broccoli (Irwin et al., 2016) and cauliflower due to a 1 bp InDel (Okazaki et al., 2007). Furthermore, a copy of *Bol.CO* was found in a small-effect QTL in the same study (Okazaki et al., 2007).

Here we provide a variant framework for flowering gene variation not only for the central flowering regulators, but for the total flowering network in both vegetable species. For example, our data could help to find functional variance for QTL in *B. rapa*, not explained by *Bra.FLC* or *Bra.FT*, in a DH population derived from the reference genotype Chiifu-401 and a rapid cycling line (Li et al., 2013). The QTL in that study on A02 and A06 may correspond to candidate genes *Bra.COL.A02* and *Bra.LFY.A06*. Although we did not detect functional variation in *Bra.LFY.A06* sequences, we identified 3 non-synonymous SNPs in *Bra.COL.A02*.

Both L58 and R-o-18 are annuals, while L58 has an early-flowering phenotype (Bagheri et al., 2012). The annual behavior has been attributed to the shared splice variant in *Bra.FLC.A10* (Yuan et al., 2009), whereas the late flowering habitus of R-o-18 could be caused by the structural variation in *Bra.FT.A07*, which seems to overlay the effect of the *Bra.FLC.A02* deletion (Zhao et al., 2010; Wu et al., 2012). A further explanation could be the duplication of *Bra.CO.A01* in L58, which is likely to increase the expression of the flowering activator CO. In contrast, the late flowering Siberian Kale Kashirka is biannual, which could either be attributed to the putative duplication of *Bol.FLC.C09b (Bo9g173400*) or to the R24Q mutation in *Bol.FLC.C03 (Bo3g024250*) and the A75D mutation in *Bol.FLC.C02* (independent mapping to *Bol.FLC.C2.E9*). All other functional variants are either shared with or unique to BRA1398, which itself is annual. *Bol.FLC.C09a* (*Bo9g173370*) seems to be a pseudogene.

All the same, the DH populations studied so far all showed transgressive segregation (Zhao et al., 2010; Bagheri et al., 2012; Xiao et al., 2013; Zhang et al., 2015), indicating polygenic regulation, so in order to identify and quantify the contributions from small effect genes, genome-wide association studies would have to complement the QTL studies performed so far. Our data represent a valuable resource for the development of suitable marker systems or for respective mutation studies.

### Rearrangements in *Brassica napus*

Most flowering time genes in *B. napus* were found to be collinear and syntenic with their orthologues in both sequenced accessions of the progenitors *B. rapa* and *B. oleracea*, in accordance to previous findings that the donor subgenomes remain basically unaltered, although local rearrangements took place (Rana et al., 2004; Parkin, 2005; Bancroft et al., 2011; Chalhoub et al., 2014). Although, the number of sequenced accessions was limited and only covered a small part of the intraspecific variation (two subspecies for *B. rapa* and one subspecies for *B. oleracea*), we do all the same believe that they give important insights into Brassica genomics. Our sequence data indicate that some copies obviously were lost from the *B. napus* genome after polyploidisation (for example, *Bol.CDF1.C02*), while others were gained by duplication (for example, *Bna.SRR1.A03*) or rearranged to another chromosome (*Bol.TFL1.C04* to *Bna.TFL1.C03*). For two gene copies among the set (*BnaCnng78500D* (*Bna.LFY.Cnn*), *BnaA02g16710D* (*Bna.ZTL.A02*), we observed a stable exchange between the subgenomes A and C in *Brassica napus*. Using resynthesized *B. napus* as a model for polyploidization, such rearrangements were observed frequently (Gaeta et al., 2007; Szadkowski et al., 2011; Schmutzer et al., 2015), and were found to occur mostly in the first meiotic cycles after hybridization (Gaeta et al., 2007). Many times, those rearrangements in resynthesized *B. napus* enclosed larger parts of a chromosome (Samans et al., 2017), whereas in our case, no consecutive patterns of fixed deletions or duplications were found, indicating small scale changes. As was found in Szadkowski et al. (2011), interspecific hybridization via unification of unreduced gametes causes more frequent, but smaller translocations than somatic doubling of allohaploids in *B. napus*. While the latter is mostly used for experimental hybrids, the first is more likely to occur under natural conditions. Small and stable homoeologous exchanges are therefore widespread in the *B. napus* genome and played a major role in *B. napus* speciation (Szadkowski et al., 2011; Chalhoub et al., 2014; Samans et al., 2017). Rearrangements (apart from CNVs) can change the regulatory context of a gene, change its mutation frequency and therefore contribute to speciation (Faria and Navarro, 2010). The occurrence of new pseudogenes both in *B. oleracea* and *B. napus* may be an indicator of beginning (*B. napus*) and ongoing (*B. oleracea*) diploidization after the interspecific hybridization. Pseudogenization and gene loss are general principles of genome evolution after whole genome duplication events (Sankoff et al., 2010). We expect that the total variation in each species will reveal even more such rearrangements. The sequence capture bait design used in the present study is therefore a valuable resource for further assessment of intra-and interspecific variation in *Brassica* flowering time genes.

## Conclusions

Flowering time control is of major importance in crop adaptation. Knowledge about flowering time genes is crucial for improving important *Brassica* vegetable crops. Our study provides sequence variation data for all orthologous copies of 35 flowering-time regulatory genes in two accessions each of *B. rapa* and *B. oleracea*, respectively. The data confirm earlier findings on variation in central flowering time regulators, but also provide comprehensive novel data spanning numerous other genes involved in the flowering network. Rearrangement patterns compared to the allotetraploid *B. napus* revealed only small and local changes, implicating that allopolyploidisation in *B. napus* occured via unreduced gametes with small-scale homoeologous exchanges.

## Author contributions

SS performed DNA extraction, bait development and data analysis. BH, DK, and RR performed library preparation and sequencing. SS and RS wrote the manuscript. All authors have read and approved the final version of the manuscript.

### Conflict of interest statement

The authors declare that the research was conducted in the absence of any commercial or financial relationships that could be construed as a potential conflict of interest.

## Supplementary material

Figure S1 Pathway variation for flowering time genes in the *B. rapa* genotype L58. Interactions are displayed as known from *Arabidopsis thaliana*. Arrows indicate positive regulation, blunt ends indicate negative regulation. All copies of a gene are displayed in a box. The main flowering regulators FT, FLC, and CO are indicated in bold boxes. The type of variant is color coded (see legend). Duplications are indicated by a second colorless copy box. SNP colors are hierarchic, meaning for example that synonymous SNPs are not displayed if non-synonymous SNPs are present. Boxes framed in red indicate known copies from *B. napus* which were not found in *B. rapa*. Boxes framed in blue were found, but not annotated as gene.

Figure S2 Pathway variation for flowering time genes in the *B. oleracea* genotype BRA1398. Interactions are displayed as known from *Arabidopsis thaliana*. Arrows indicate positive regulation, blunt ends indicate negative regulation. All copies of a gene are displayed in a box. The main flowering regulators FT, FLC, and CO are indicated in bold boxes. The type of variant is color coded (see legend). Duplications are indicated by a second colorless copy box. SNP colors are hierarchic, meaning for example that synonymous SNPs are not displayed if non-synonymous SNPs are present. Boxes framed in red indicate known copies from *B. napus* which were not found in *B. oleracea*. Boxes framed in green indicate that this copy was not known from *B. napus*. Boxes framed in blue were found, but not annotated as gene.

Table S1 Total number of target SNPs and number of homozygous target SNPs for both sequenced species along with the number of target SNPs and homozygous target SNPs for each sequenced genotype.

Table S2 Total number of target InDels and number of homozygous target InDels for both sequenced species along with the number of target InDels and homozygous target InDels for each sequenced genotype.
