# Supplementary figures and images for: Flowering Time Gene Variation in Brassica Species Shows Evolutionary Principles
Source: Front Plant Sci. 2017 Oct 17;8:1742. doi: 10.3389/fpls.2017.01742 (PMC5651034; doi:10.3389/fpls.2017.01742)

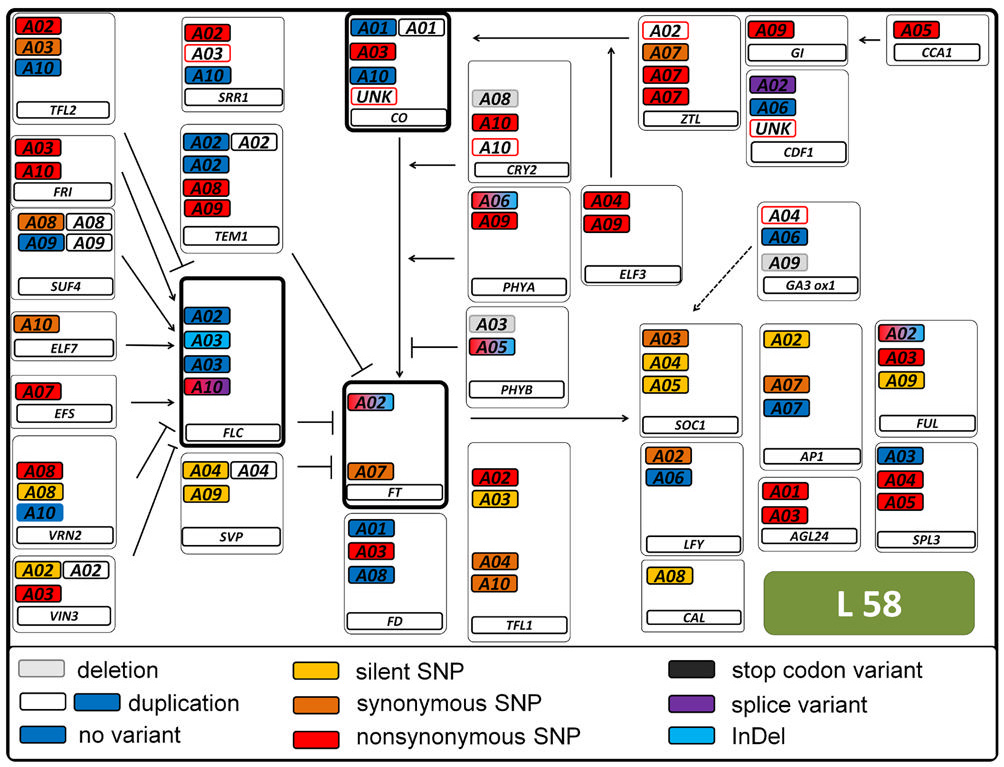

Supplement: Figure S1 — Pathway variation for flowering time genes in the B. rapa genotype L58. Interactions are displayed as known from Arabidopsis thaliana. Arrows indicate positive regulation, blunt ends indicate negative regulation. All copies of a gene are displayed in a box. The main flowering regulators FT, FLC, and CO are indicated in bold boxes. The type of variant is color coded (see legend). Duplications are indicated by a second colorless copy box. SNP colors are hierarchic, meaning for example that synonymous SNPs are not displayed if non-synonymous SNPs are present. Boxes framed in red indicate known copies from B. napus which were not found in B. rapa. Boxes framed in blue were found, but not annotated as gene. [file Image1.TIF]

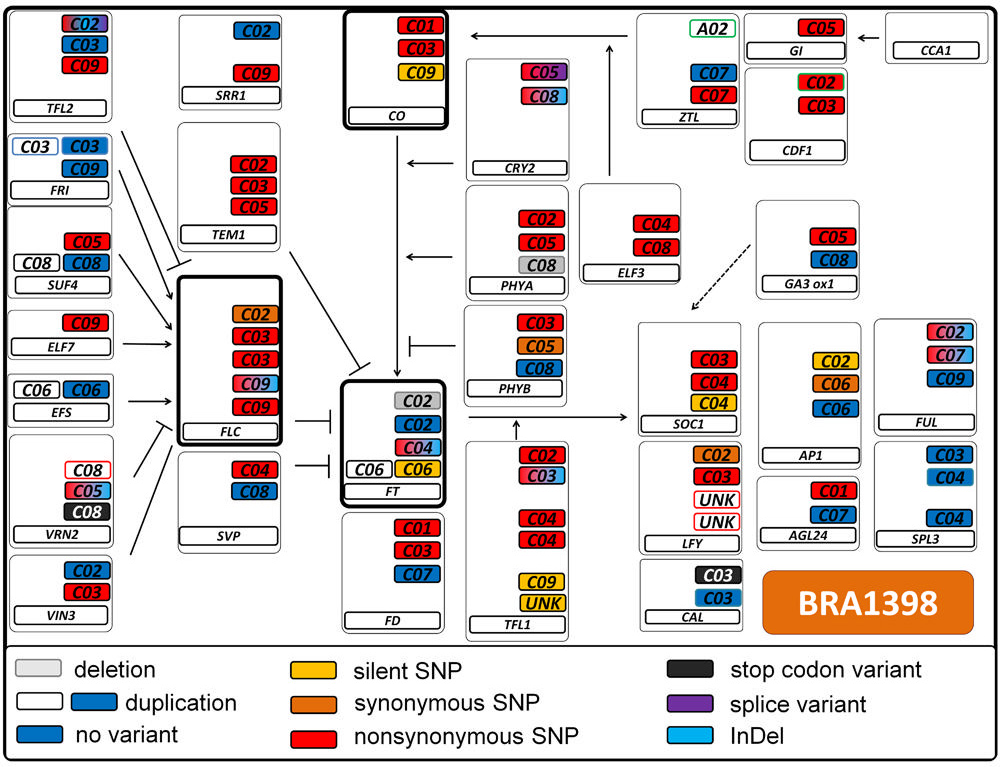

Supplement: Figure S2 — Pathway variation for flowering time genes in the B. oleracea genotype BRA1398. Interactions are displayed as known from Arabidopsis thaliana. Arrows indicate positive regulation, blunt ends indicate negative regulation. All copies of a gene are displayed in a box. The main flowering regulators FT, FLC, and CO are indicated in bold boxes. The type of variant is color coded (see legend). Duplications are indicated by a second colorless copy box. SNP colors are hierarchic, meaning for example that synonymous SNPs are not displayed if non-synonymous SNPs are present. Boxes framed in red indicate known copies from B. napus which were not found in B. oleracea. Boxes framed in green indicate that this copy was not known from B. napus. Boxes framed in blue were found, but not annotated as gene. [file Image2.TIF]
